# Acknowledgment to the Reviewers of *Neurology International* in 2022

**DOI:** 10.3390/neurolint15010010

**Published:** 2023-01-19

**Authors:** Neurology International Editorial Office

**Affiliations:** MDPI AG, St. Alban-Anlage 66, 4052 Basel, Switzerland

High-quality academic publishing is built on rigorous peer review. *Neurology International* was able to uphold its high standards for published papers due to the outstanding efforts of our reviewers. Thanks to the efforts of our reviewers in 2022, the median time to first decision was 20 days and the median time to publication was 44 days. Regardless of whether the articles they examined were ultimately published, the editors would like to express their appreciation and thank the following reviewers for the time and dedication that they have shown *Neurology International*:
Adnan KhanKeun-Yeong JeongAgata StanekKore Kai LiowAlessandro BurlinaLászló VécseiAlexandre González-RodríguezLilach SoreqAnastasia BougeaLouis H. WeimerAnastasiya E. RunnovaLuigi Alberto PiniAnca ZanfirescuŁukasz A. PoniatowskiAndrea AlexandreLydia Giménez-LlortAngel Morales-GonzálezMahesh RachamallaAngelo TorrenteManabu NatsumedaAnil Kumar JonnalagaddaManuela LeriAnthony L. KomaroffMarco GermanottaAntonietta BernardoMarek FraněkAntonio AmmendoliaMaria SkokouAntonio LeoMariagiovanna CantoneBeatrice CairoMarta MonzónBianca E. ŐszMarzia SegùBrajesh SinghMasaru TanakaCaroline SpeksnijderMatteo FoschiCelina WojciechowskaMaurizio EliaChiaki YokotaMehdi GhasemiChian Ju JongMelanie WalkerChristian TanislavMickael BonnanCristian DeanaMiren AltunaDaniel López-LópezMirza PojskicDaniela CalinaMonika Les̈kiewiczDaniela Carmen AbabeiMuthu ThiruvengadamDaniela HaluzaNatalia A. MuralevaDaniil P. AksenovNataliya KolosovaDario CalafioreNeil McGregorDhanesh Sivadasan BinduNicola MontemurroDimitrios KasselimisNicola MumoliDipak RanaNicola ValèDmitriy ShcherbakovNicolae GicaDmitry E. PostnovNicole CoufalDragana ProticNoriaki TomuraDragos Catalin JianuOmar El HibaDumitru CiolacPablo HervellaElena R. LebedevaPaolo CampioniEleonora VirgilioPaul WillnerElisabetta BertelliniPaulius GrigaravičiusEliyahu DremencovPaweł WańkowiczEmanuela EspositoPeng ChenEmilia M. GattoPeter KarachunskiEmmanouil V. DermitzakisPeter KoulenEnquan XuPierre DuquetteEraldo OcchettaPing-Tao TsengErmelinda De MeoRaffaele FrazziEva BagyinszkyRaffaele OrnelloEwa Gibuła-TarłowskaRainer LeonhartFabrice DarcheRajesh K. TripathyFiras KobeissyRajkumar VutukuriFrancesca FerroniRedi RahmaniFrancesca ManniRodica BălașaFrancesco CoreaRomeo DobrinFrancesco OrziSalvatore IaconoFrancisco Guillen-GrimaSalwa El TawilFrank WeberSamuel ShiFrederik DenormeSara ColloroneGeorgia KaiafaSebastian SchnaubeltGhulam Md AshrafSeon-Cheol ParkGianmarco de DonatoShancheng BaoGiovanna BorrielloShazid Md. SharkerHansen DengShinji KawabataHarold L. RekateSilvana AlfeiHassan BanarueeSimone BattagliaHidetoshi KomatsuSireesha ManneHongsun GuoSofya P. KulikovaHorea G. RusStephanie DuguezHueng-Chuen FanSuman ChowdhuryIdolo TedescoTakeshi KondohIgor Y. IskusnykhTatiana AntipovaIneke Van Der BurgtThomas M. FreimanIoannis LiampasThomas WeltonIsmini E. PapageorgiouTomasz WolnyIzabela ZakrockaTommaso ErcoliJaclyn Hennessey FordTommaso ZoerleJan LehotskyTomohiko SadaokaJan NovákTomohiro ToriiJan PithaTudor PopJasmina IsakovićTurcu-Stiolica AdinaJin Hyuck ParkVijai KrishnanJinming HanVincent PonsJoan BagóVincenzo Di StefanoJoana DamásioWeihua DingJoanna SikoraXinxing WangJohann SellnerXueying WangJunji YamauchiYang HeKabirullah LutfyYi‐Chia WeiKah-Hui WongYu ZhangKarolina NoworytaYujiao LuKatarzyna KaczyńskaYuki MiyamotoKavita SinghZbârcea Cristina ElenaKeld-Erik Byg



